# Impact of palliative care at end-of-life Covid-19 patients – a small-scale pioneering experience

**DOI:** 10.1186/s12904-024-01368-9

**Published:** 2024-02-10

**Authors:** João Luís Rodrigues-Ribeiro, Luísa Castro, Filipa Pinto-Ribeiro, Rui Nunes

**Affiliations:** 1 Palliative Care Unit, WeCare Saúde, Rua Corregedor Gaspar Cardoso, 480, Póvoa de Varzim, Porto, 4490-492 Portugal; 2 Faculty of Medicine, University of Porto, Porto, 4200-319 Portugal; 3 Center for Health Technology and Services Research (CINTESIS), Faculty of Medicine, University of Porto, Porto, 4200-319 Portugal; 4 Department of Community Medicine, Information and Health Decision Sciences (MEDCIDS), Faculty of Medicine, University of Porto, Porto, 4200-319 Portugal; 5 Life and Health Sciences Research Institute (ICVS), School of Medicine, University of Minho, Braga, 4710-057 Portugal; 6 ICVS/3B’s-PT Government Associate Laboratory, Guimarães, 4806-909 Portugal; 7 Intra-Hospital Team for Palliative Care Support, Hospital de Braga, ULS Braga, Braga, Portugal

**Keywords:** Palliative care, End of life, Covid-19, Symptom control, Deprescription

## Abstract

**Background:**

In March 2020, the outbreak caused by the SARS-CoV-2 virus was declared a pandemic, resulting in numerous fatalities worldwide. To effectively combat the virus, it would be beneficial to involve professionals who specialize in symptom control for advanced illnesses, working closely with other specialties throughout the illness process. This approach can help manage a range of symptoms, from mild to severe and potentially life-threatening. No studies have been conducted in Portugal to analyse the intervention of Palliative Medicine at the end of life of Covid-19 patients and how it differs from other specialties. This knowledge could help determine the importance of including it in the care of people with advanced Covid-19.

**Objectives:**

The objective of this study is to examine potential differences in the care provided to patients with Covid-19 during their Last Hours and Days of Life (LHDOL) between those who received care from Palliative Medicine doctors and those who did not.

**Methods:**

This is a retrospective cohort study spanning three months (Dec 2020 to Feb 2021), the duration of the Support Unit especially created to deal with Covid-19 patients. The database included clinical files from 181 patients admitted to the Support Unit, 27 of which died from Covid-19.

**Results:**

Statistically significant differences were identified in the care provided. Specifically, fewer drugs were administered at the time of death, including drugs for dyspnoea, pain and agitation, suspension of futile devices and use of palliative sedation to control refractory symptoms.

**Conclusions:**

End-of-life care and symptomatic control differ when there’s regular follow-up by Palliative Medicine, which may translate less symptomatic suffering and promote a dignified and humane end of life.

## Background

The Covid-19 pandemic is caused by the severe acute respiratory syndrome coronavirus 2 (SARS-CoV-2) [1]. It was first identified during an outbreak in Wuhan, China in December 2019 [2]. Despite various containment attempts, the virus spread to other areas of China and subsequently around the world [3]. On 11 March 2020, the World Health Organisation (WHO) declared a pandemic, prompting countries to implement preventive measures [4, 5]. As of 9 May 2023, WHO has reported over 760 million confirmed cases and 6.9 million deaths globally. In Portugal, WHO has reported over 5.5 million confirmed cases and 26,600 deaths as of 16 April 2023. Worldwide, 13.3 billion vaccine doses have been administered [6].

### Palliative care

Palliative care is a form of care that seeks to improve the quality of life for patients and their families who are facing issues related to an incurable and/or serious illness. This involves identifying and addressing physical, psychosocial, and spiritual issues early on to alleviate suffering. Professionals in palliative care regard life as valuable and death as a natural process that should not be hastened or delayed. It could be argued that there is a universal right to access palliative care [7].

Palliative care provides a support system that integrates psychological and spiritual care components to help patients live as actively as possible until death. Additionally, it assists families in coping with their loved one’s illness and bereavement. The intervention is based on interdisciplinarity [8].

In Portugal, the recognition of a Medical Competence in Palliative Medicine is a recent development. Currently, 104 doctors across the country hold this qualification, having undergone intensive practical and theoretical training to meet all the necessary requirements [9].

In 2020, the *Clínica Universitaria de Navarra* published guidelines for the palliative medicine approach in Covid-19 patients. The document acknowledges that dyspnoea, fever, agitation, pain and bronchorrhea are the most prevalent symptoms in advanced disease [10, 11].

The severity of symptoms associated with the disease can vary depending on its progression, ranging from easily manageable to highly painful [12–16].

If symptoms do not respond to optimised therapeutic protocols, they are referred to as refractory symptoms. At this point, palliative sedation may be considered [17].

It is important to clarify the concept behind the technique of intentional reduction of consciousness, known as Palliative Sedation, as there is no universal definition for it. This procedure is used to control intolerable suffering caused by symptoms that are refractory to conventional treatment in the terminal phase of incurable and progressive diseases [18, 19]. This definition excludes subjective evaluations unless clearly marked as such. Palliative sedation is a practice employed by Palliative Medicine Services for eligible patients, and the assessment of these professionals is crucial in making the decision.

Managing patients with chronic and progressive diseases who are taking multiple medications is a recognised challenge, including the issue of therapeutic futility [20]. In medical practice, recommendations for primary or secondary prevention often suggest initiating therapy, but there are few indications for simplifying treatment [21]. Therapeutic simplification, also known as ‘deprescribing’, optimises a patient’s therapeutic regime by discontinuing inappropriate or unnecessary drugs and considering the individual care plan [22, 23]. Studies indicate that inappropriate medication use is frequent in situations of terminal illness [24, 25], prompting us to question our actions in situations where ‘less can be more’.

### Objectives

The aim of this study was to assess and determine whether there are any differences in the care provided to patients admitted in a support inpatient unit who were followed by palliative care doctors compared to patients who were not.

## Materials and methods

### Framework

The increase in Covid-19 cases has required health services to collaborate in order to expand and meet the demands for care. Consequently, field hospitals, support hospitals, and support units have been established [26, 27].

The Narciso Ferreira Hospital is a medical facility owned by Santa Casa da Misericórdia de Riba D’Ave, a town in the Portuguese municipality of Vila Nova de Famalicão, with an area of 2.83 km² and a population of 3,425 [28]. Between December 2020 and February 2021, an agreement was made with ARS Norte (Northern Regional Health Administration) to establish a support unit for Covid-19 patients, resulting in an additional 20 beds being made available.

The new unit has formed a multidisciplinary team consisting of doctors from General Practice and Family Medicine, Internal Medicine, and Emergency Medicine. Furthermore, Palliative Medicine doctors have been included as an essential part of the team responsible for Covid-19 patients, rather than just as support if necessary. Patient distribution is random and depends on the clinicians on duty each day. When a doctor specialising in Palliative Medicine is on duty, they are assigned to patients who are in an advanced stage. These patients have already been identified in their hospital of origin as not being candidates for Advanced Life Support (ALS) in the event of cardio-respiratory arrest, as well as patients who are considered to be in a terminal phase. Terminal patients were classified, although controversial, as such if it would not be a surprise if the patient died within 6 months [29]. Additionally, it was also the palliative care specialist who categorized a patient as in LHDOL after admission. It should be noted LHDOL is a clinical diagnosis, also denominated as Imminent Death Syndrome and consists of a set of signs/symptoms that generally appear in 3 stages, as follows [30]:

Initial:

- Bedridden.
- Loss of interest or ability to eat/hydrate.
- Cognitive changes: increased sleep time, delirium.

Intermediate.

- Further decline in mental state until obtundation (slow response to stimuli, short periods of wakefulness).

Late:

- Terminal bronchorrhea (secretions accumulate in the oral cavity which are not mobilised due to loss of the swallowing reflex).
- Coma.
- Fever.
- Altered breathing pattern (Cheyne-Stokes pattern, alternating between hyperpnoea and periods of apnoea).
- Spots on the extremities.

In this way, it was guaranteed that all members of the medical team, whether palliative care specialist or not, had complete knowledge of the current status of their patient.

### Study setting and sampling

This is a retrospective observational cohort study that analysed a sample of patients who passed away in a support hospital in northern Portugal which was in operation between 2nd December 2020 and 28th February 2021. The study was approved by the Ethics Committee of the Narciso Ferreira Hospital (CES-SCMRA 007/2021). The data was stored in an Excel database created by the researcher, and only the researcher had access to it. The study analysed all deaths that occurred in the support hospital and followed the STROBE guidelines for observational cohort studies. The deaths were categorised into two groups based on their follow-up at the LHDOL: patients who received care from Palliative Medicine doctors (PM Group) and patients who received care from doctors in other specialties (OS Control Group), as determined by consulting the Sclinico files (the hospital’s intranet system). The study analysed the following variables: age, number of drugs prescribed during hospitalisation until the time of death, medical devices used such as O2, nasogastric tube, and bladder tube and use of palliative sedation.

### Statistical analysis

The statistical analysis of the variables was conducted using IBM SPSS Statistics 27 software. Descriptive analysis of the data was performed using absolute and relative frequencies for categorical data, and median with interquartile range (IQR) for quantitative variables that were identified as not normally distributed by visualising the histogram. The variables were compared between the two groups using the Mann-Whitney test. The Chi-squared test or Fisher’s exact test (in cases where at least 20% of the expected absolute frequencies were less than 5) was used to compare categorical variables between the groups. A significance level of 0.05 was set *a priori* to identify statistically significant differences.

## Results

**Fig. 1:**
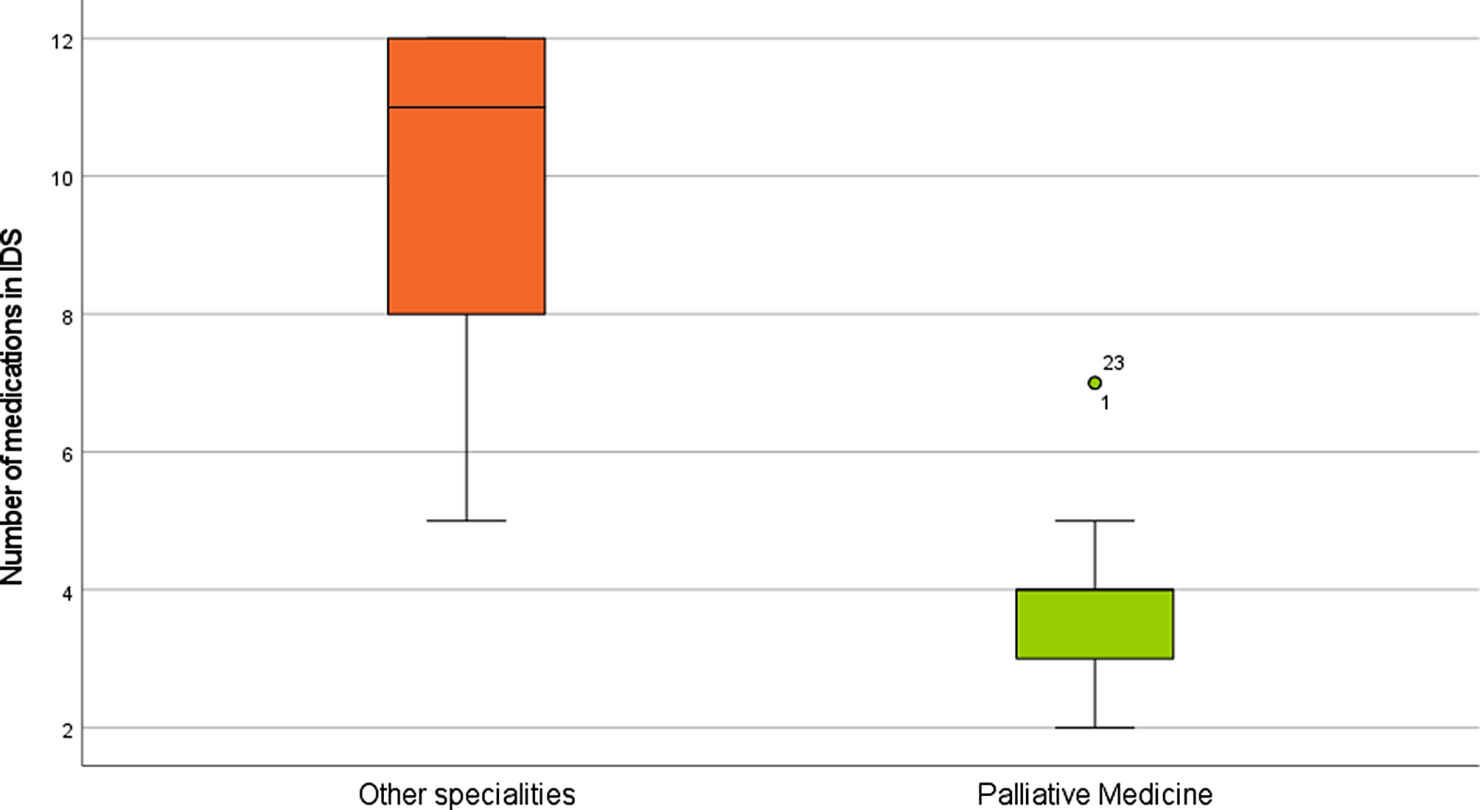
Comparison of the number of medications in the Imminent Death Situation (IDS) between groups of patients followed by Palliative Medicine doctors (*n* = 13) and those followed by other specialties (*n* = 14)

During the study period, 181 patients were admitted to the support unit, and 27 of them passed away. The median age of the patients was 84 years (ranging from 55 to 96). Out of these patients, 13 were followed up by Palliative Medicine, with a median age of 83 years (ranging from 69 to 89). The remaining 14 patients, with a median age of 84 years (ranging from 55 to 93), were not followed up. According to Fig. 1, the group monitored by palliative care doctors took significantly less medication than the group that was not monitored (median [IQR] = 11[8;12] vs. 4[3;4.5], respectively; *p* < 0.001).

In relation to basic/preventive medication for controlling expected end-of-life symptoms, the group followed by palliative care specialist had significantly higher usage rates for symptoms such as agitation (76.9% vs. 28.6%; X2(1) = 6.312; *p* = 0.006) and pain (100% vs. 42.9%; *p* = 0.001). There was no significant difference between the two groups in terms of dyspnoea (100% vs. 85.7%; *p* = 0.481), fever (30.8% vs. 14.3%; *p* = 0.385), and secretions (40.2% vs. 14.3%; *p* = 0.103) in the proportion of patients medicated.

**Fig. 2:**
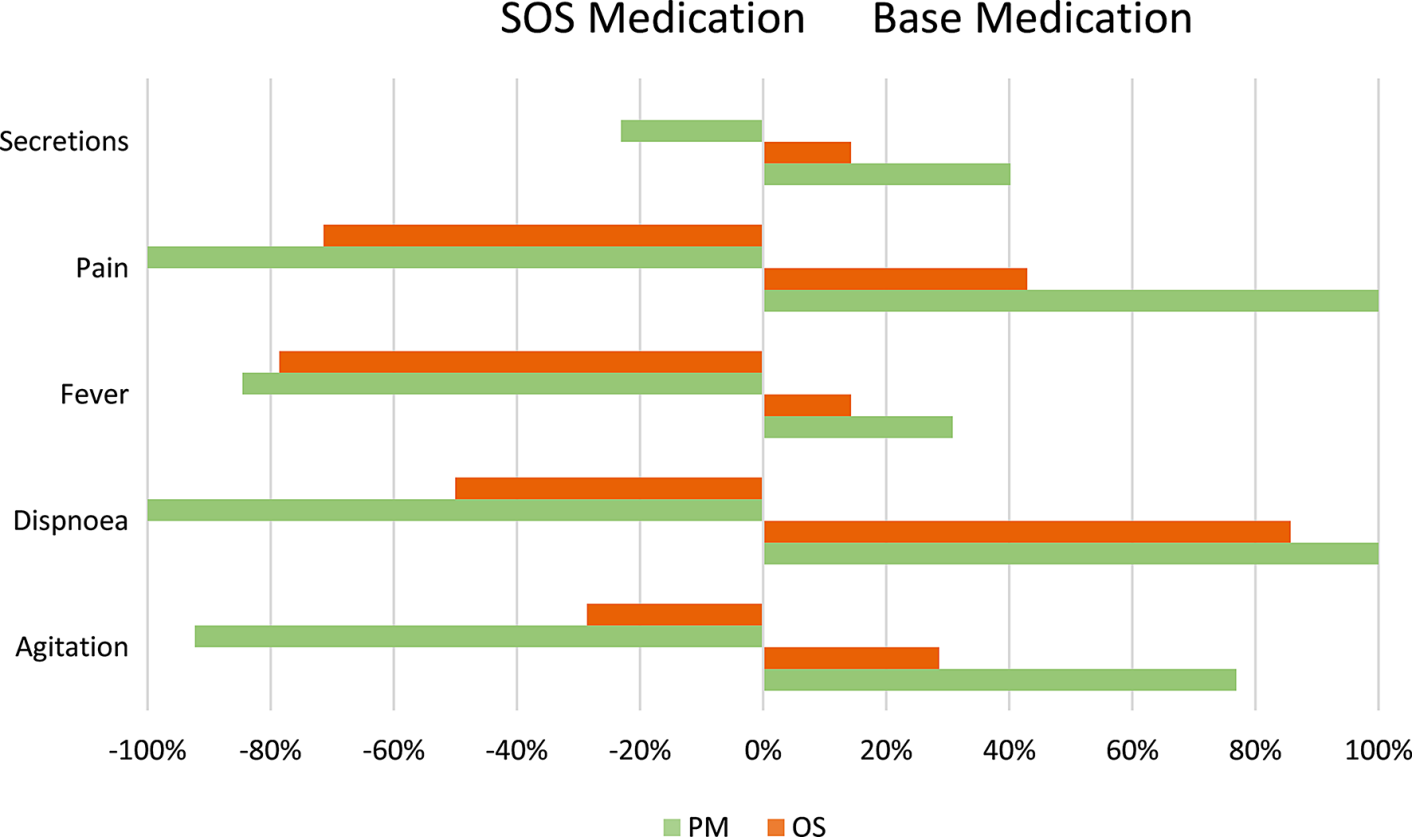
Comparison between basic medication and SOS medication by symptom for both groups

Statistically significant differences were found between the two groups for agitation (92.3% vs. 28.6%; X2(1) = 11.342; *p* < 0.001) and dyspnoea (100% vs. 50%; *p* = 0.004) regarding the immediate prescription of SOS (rescue medication) upon admission, with the aim of using it immediately in the event of decompensation. However, there were no significant differences found in the use of rescue medication for fever (84.6% vs. 78.6%; *p* = 1.000), pain (100% vs. 71.4%; *p* = 0.98), and secretions (23.1% vs. 0.0%; *p* = 0.98) (see Fig. 2).

As for therapeutic reconciliation, the proportion was significantly higher in the palliative care group (100% vs. 7.1%; X2(1) = 23.281; *p* < 0.001).

The reason for using supplementary oxygen was also evaluated, and it was found that the proportion of patients with oxygen to target saturations was significantly higher in the group not followed by palliative care (78.6% vs. 30.8%; X2(1) = 6.238; *p* = 0.006).

The study examined the decision to simplify measurements by removing devices. If oxygen did not provide symptomatic benefit, the proportion of patients whose support was deemed futile and consequently removed was significantly higher in the palliative care group (53.8% vs. 0%; *p* = 0.003). A statistically significant difference was observed in the decision to withdraw NGT (nasogastric tubes) in LDHOL situations (100% vs. 0%, *p* < 0.001). Similar results were obtained for urinary catheterisation.

The study also analysed the use of palliative sedation, which was found to be necessary for 53.8% of patients due to refractory symptoms. Our data revealed that the proportion of patients who received palliative sedation at the end of life was significantly higher in the palliative care group (53.8% vs. 0%; *p* = 0.002).

## Discussion

Our data suggests the approach to a patient in LHDOL is different depending on the previous training of the clinician which is accordingly reflected in the therapeutical plan drawn up for their patient. Palliative Care Specialists opt for a more holistic approach, essential for a complete and individualized care [31, 32]. It marks the transition from the healing process to the caring process.

It is worth noting that many professionals may find it challenging to achieve therapeutic reconciliation or simplification [20; 22–25]. Defining which drugs are futile and which ones will actually bring some benefit to the patient, can lead to discussion and the need for meetings with family members and/or other professionals to clarify the changes in the therapeutical approach. In fact, involving patients, family members and other professionals in the process can be beneficial for a better understanding of the outcomes [33–35].

Palliative Medicine Physicians are trained in a thoughtful and balanced exercise of pharmacological therapy to use all the potential of each single drug. This approach comprises utilizing side effects and potential interactions to achieve the desired effect. The goal is to do the most with the least, in a stage where extending life or preventing disabilities becomes secondary [36] and the major outcome becomes the best quality of life possible with the less drugs possible and avoid polypharmacy [37–40].

Our study demonstrated a significant difference in the number of drugs prescribed in the LHDOL between patients who received a palliative approach compared to those who did not. Given the context, during the pandemic, this disparity could be explained by limited human resources, differences in background training, LHDOL survival prognostication or follow up by palliative care specialists, all of which are in line with existing literature [41–46], and could be grouped in two barrier types: organizational and professional, as suggested by Paque et al. [43].

Futility or therapeutic zeal can become a slippery slope, either by the professionals themselves, through their desire and commitment to “not give up” on the patient, or through the pressure of family members or carers who, often, if they are not properly informed, can create resistance to discontinuing a drug that the patient has taken throughout their lives for diseases such as hypertension or diabetes [47]. Perhaps comparing the economic burden of palliative and non-palliative approaches on the management of LHDOL patients at the health care and individual levels and correlating it with gain in quality of life would prove beneficial to guide both health care professionals and family members or caretakers in their decision making.

Covid-19, like any disease, presents characteristic signs and symptoms that provide essential clues for a correct diagnosis. The palliative medicine approach aims not only to control existing symptoms, but also to prevent any potential suffering of the patient by administering basal and controlled doses of drugs, assessed on a case-to-case base, that can even prevent symptoms from appearing [8]. Per example, the importance of palliative care intervention in tracheostomized Covid-19 patients is consensual although there are still lack of guidelines for this cooperation [48].

We observed no difference in the baseline administration of drugs for dyspnoea and fever between the two groups, while there was a significant difference in baseline drugs for agitation, as these were more frequently not prescribed by non-palliative care professionals. This difference in the management of agitation is particularly worrisome due to the high prevalence of this symptom in Covid-19 patients in LHDOL [48–51]. It should be noted that certain drugs, particularly benzodiazepines, can be used to manage dyspnoea as well as agitation, by taking advantage of their double effect [52, 53].

By contrast, when evaluating the prescription of SOS drugs by Support Unit professionals, our data found statistically significant differences in agitation and dyspnoea, but not in fever, pain, and secretions. In Palliative Medicine, physicians often choose drugs based on their effects on predictable acute suffering resulting from decompensation of the underlying disease [54]. Systematic search and treatment of the most frequent symptoms is crucial for patient management, but prevention should also be prioritised. However, and although the most commonly reported symptoms in the last days of life of Covid-19 patients are agitation, dyspnoea, fever, pain and secretions [55–60], non-palliative specialists do not appear to adjust their prescriptions aiming at prevention. In the future, it would be beneficial to evaluate specific drugs and their intended purpose as well as the impact of training of non-palliative professionals in their use in specific contexts.

Another difference between Covid-19 in LHDOL patient management between palliative and non-palliative professionals was observed in the use of medical devices, with the former resourcing less often to their use. Oxygen therapy, largely used in ICU Covid-19 inpatients [61], solely for the purpose of achieving target oxygen saturation levels, at the expense of user comfort, should not be considered an effective treatment [62, 63]. While supplemental oxygen therapy should be used only to improve comfort by addressing signs and symptoms, it should not be used to achieve target saturations without regard for the individual’s current clinical condition. The philosophy of Palliative Medicine is to treat people rather than numbers. Therefore, the use of oxygen will primarily relieve dyspnoea rather than achieving normal saturation levels observed on a saturation meter [64]. In some cases, this difference may even justify the decision to stop oxygen therapy permanently [61]. In this context, decisions relating to: (1) the withdraw of nasogastric tubes (NGTs) should be carefully considered, since its usefulness in the management of patients in LDHOL is questionable [65–70]; (2) resourcing to subcutaneous route in order to route rotation and avoid unnecessary venous catheterisation, should be prioritised [71], and; (3) bladder catheterisation should be avoided due to increased risk of discomfort and infection [72–74]. It is important to highlight that professionals in Palliative Medicine receive specific training in recognizing IDS signs [75], which can aid in decision-making, particularly regarding device removal.

As expected, palliative sedation was significantly higher in patients followed by palliative care specialists, probably due to their experience in the use of drugs to preserve well-being while managing the level of consciousness. Our results differ from those of Ramos-Rincon et al. [51] as in their work palliative sedation was also administered by non-palliative specialists, however it is not possible to explore this disparity as the authors did not examine this subject.

Despite our findings, it is important to acknowledge certain limitations in this work. The study was conducted during the Covid-19 outbreak, but it is important to note that the pandemic is ongoing. Palliative medicine and symptom control are valuable not only for managing Covid-19, but also for addressing other diseases and conditions that can significantly impact quality of life. The study’s sample size was limited not only due to the short three-month duration of the support unit, but also to the unique nature of this support unit care which integrated a Palliative Medicine Specialist as part of the primary team. Consequently, the findings may not be representative of the entire population. Importantly, the fact that our data didn’t include the assessment of symptoms or quality of life is also a limitation, because these evaluations are the primary aim of palliative care and not the removal of devices, reduction of medication or lowering of consiousness as might have transpired from our assessment. On the other hand, these latter decision are an important and controversial points of care that need to be discussed and clarified when working in a multidisciplinary team. Finally, the study’s retrospective nature, based on clinical records, may have resulted in less accurate findings. Further and larger studies are required to validate these exploratory results. Therefore, we hope that this study can serve as a catalyst for improvement of palliative patients and future research in this field.

## Conclusion

The main target of Palliative Care will always be providing the best comfort and dignity to our patients, using only interventions that really contribute to target it. Prolonging life with the aid of devices and polymedication is not part of good practices in caring for the terminally ill. The comparison between measures undertaken, with or without specialists, in palliative care showed that these were less frequent in palliative care specialists. A team specialising in Palliative Medicine could bring significant benefits if it were to participate actively in the front line, rather than just as consultants. Death is a challenging process, and every individual should have the right to a dignified death that honours the life they lived. Investing in training in Palliative Medicine could bring long-term benefits in dealing with patients at the end of life and their families, leading to a more dignified end for each individual. This could also alleviate suffering in Covid-19 patients through targeted therapies and individualised care.

## Data Availability

Upon request, the corresponding author can provide the datasets used and/or analysed in the current study.

## Abbreviations

ALS: Advanced Life Support
IDS: Imminent Death Situation
LHDOL: Last Hours or Days of Life
NGT: Nasogastric Tube
WHO: World Health Organization

## References

[1] Grolli RE, Mingoti ME, Bertollo AG (2021). Impact of COVID-19 in the mental health in elderly: psychological and biological updates. Mol Neurobiol.

[2] Merad M, Blish CA, Sallusto F, Iwasaki A (2022). The immunology and immunopathology of COVID-19. Science.

[3] Khan M, Adil SF, Alkhathlan HZ (2020). COVID-19: A global challenge with Old History, Epidemiology and Progress so far. Molecules.

[4] WHO Director-General’s opening remarks at the media briefing on COVID-19, 11. March 2020, available from https://www.who.int/director-general/speeches/detail/who-director-general-s-opening-remarks-at-the-media-briefing-on-covid-19---11-march-2020, accessed on 21 August 2022.

[5] Wong SYS, Zhang D, Sit RWS (2020). Impact of COVID-19 on loneliness, mental health, and health service utilisation: a prospective cohort study of older adults with multimorbidity in primary care. Br J Gen Pract.

[6] World Health Organization. 2023, WHO Coronavirus (Covid-19) Dashboard, available from https://covid19.who.intint accessed on 11 May 2023.

[7] Healthcare as a Universal Human Right (2022). Sustainability in Global Health, Rui Nunes.

[8] Radbruch L, Redefining Palliative Care-A New Consensus-Based Definition (2020). J Pain Symptom Manage.

[9] Ordem dos Médicos. 2023, website da Ordem dos Médicos, available from https://ordemdosmedicos.pt/criterios-de-admissao-na-competencia-em-medicina-paliativa accessed on 23 May 2023.

[10] Clinica Universidad de Navarra 2021, Guías rápidas de apoyo y control sintomático en pacientes avanzados con COVID-19. Available from https://cuidadospaliativos.org/blog/wp-content/uploads/2020/03/Guia-COVID-19.V.2.0_22.3.20.pdf, accessed on 23 July, 2022.

[11] Wong AK, Demediuk L, Tay JY (2021). COVID-19 end-of-life care: symptoms and supportive therapy use in an Australian hospital. Intern Med J.

[12] Wiersinga WJ, Rhodes A, Cheng AC, Peacock SJ, Prescott HC (2020). Pathophysiology, transmission, diagnosis, and treatment of Coronavirus Disease 2019 (COVID-19): a review. JAMA.

[13] Mao L, Jin H, Wang M (2020). Neurologic manifestations of hospitalized patients with Coronavirus Disease, 2019 in Wuhan, China. JAMA Neurol.

[14] Chen YT, Shao SC, Hsu CK, Wu IW, Hung MJ, Chen YC (2020). Incidence of acute kidney injury in COVID-19 infection: a systematic review and meta-analysis. Crit Care.

[15] Mao R, Qiu Y, He J-S (2020). Manifestations and prognosis of gastrointestinal and liver involvement in patients with COVID-19: a systematic review and meta-analysis. Lancet Gastroenterol Hepatol.

[16] Long B, Brady WJ, Koyfman A, Gottlieb M (2020). Cardiovascular complications in COVID-19. Am J Emerg Med.

[17] Juth N, Lindblad A, Lynöe N, et al. European Association for Palliative Care (EAPC) framework for palliative sedation: an ethical discussion. BMC Palliat Care. 2010;20. 10.1186/1472-684X-9-20. 9,:. 10.1186/1472-684X-9-20 PMC2945325 20836861

[18] Katherine Morrison MD, Jeanie Youngwerth MD (2016). - Palliat Sedation Therapy Hosp Med Clin.

[19] Fabiola Leite (2012). Nogueira et al- palliative sedation of terminally ill patients. Rev Bras Anestesiol.

[20] Davies E, Higginson IJ, editors. Palliative care. The solid facts. Geneva: World Health Organization; 2004. 32 p. available at http://www.euro.who.int/document/E82931.pdf accessed on 12 January 2023.

[21] Kutner JS, Blatchford PJ, Taylor DH, Christine S, Bull JH (2015). Safety and Benefit of discontinuing statin therapy in the setting of Advanced, Life-limiting illness: a Randomized Clinicaln Trial. JAMA Intern Med.

[22] Akinbolade O, Husband A, Forrest STA (2016). Deprescribing in advanced illness. Prog Palliat Care.

[23] Thompson W, Farrell B, Deprescribing. What is it and what does the evidence tell us? Can J Hosp Pharm. 2013;201–2. 10.4212/cjhp.v66i3.1261 PMC3694945 23814291

[24] Riechelmann RP, Krzyzanowska MK (2009). Futile medication use in terminally ill cancer patients. Support Care Cancer.

[25] Romero I (2018). Desprescrever nos doentes em Fim De Vida: um guia para melhorar a Prática Clínica. Rev Port Soc Med Interna.

[26] Diário da República Eletrónico. 2022, website do Diário da República, Despacho n.º 10942-A/2020, de 6 de novembro, available at https://dre.pt/dre/detalhe/despacho/10942-a-2020-147814594, accessed on 24 March 2023.

[27] de Serviço Nacional. Saúde 2023, website do Serviço Nacional De Saúde, Plano-da-saúde-para-o-outono-inverno-2020-21, available at www.sns.gov.pt, accessed on 24 February 2023.

[28] Junta de Freguesia de Riba D’Ave. 2022, website da Junta de Freguesia de Riba D’Ave, available from https://www.jf-ribadeave.pt/riba-de-ave/, accessed on 22 October 2022.

[29] Hui D, Nooruddin Z, Didwaniya N (2014). Concepts and definitions for actively dying, end of life, terminally ill, terminal care, and transition of care: a systematic review. J Pain Symptom Manage.

[30] Alsuhail AI, Punalvasal Duraisamy B, Alkhudhair A, Alshammary SA, AlRehaili A (2020). The Accuracy of Imminent Death diagnosis in a Palliative Care setting. Cureus.

[31] Hackett J (2017). The Importance of Holistic Care at the end of life. Ulster Med J.

[32] Greer S, Joseph M, Palliative Care (2016). A holistic Discipline. Integr Cancer Ther.

[33] Mortsiefer A, Löscher S, Pashutina Y (2023). Family conferences to facilitate deprescribing in older outpatients with Frailty and with polypharmacy: the COFRAIL Cluster Randomized Trial. JAMA Netw Open.

[34] Powazki R, Walsh D, Hauser K, Davis MP (2014). Communication in palliative medicine: a clinical review of family conferences. J Palliat Med.

[35] Bångsbo A, Dunér A, Lidén E. Patient participation in discharge planning conference. Int J Integr Care. 2014;14(e030). 10.5334/ijic.1543. 10.5334/ijic.1543 PMC4236306 25411572

[36] Holmes H (2009). Rational prescribing for patients with a reduced life expectancy. Clin Pharmacol Ther.

[37] Cruz-Jentoft AJ, Boland B, Rexach L (2012). Drug therapy optimization at the end of life. Drugs Aging.

[38] Lee HR, Yi SY, Kim DY (2013). Evaluation of prescribing medications for terminal Cancer patients near death: essential or futile. Cancer Res Treat.

[39] Kierner KA, Weixler D, Masel EK, Gartner V, Watzke HH (2016). Polypharmacy in the terminal stage of cancer. Support Care Cancer.

[40] Currow DC, Stevenson JP, Abernethy AP, Plummer J, Shelby-James TM (2007). Prescribing in palliative care as death approaches: polypharmacy in palliative care. J Am Geriatr Soc.

[41] Boddaert MS, Stoppelenburg A, Hasselaar J, van der Linden YM, Vissers KCP, Raijmakers NJH (2021). Specialist palliative care teams and characteristics related to referral rate: a national cross-sectional survey among hospitals in the Netherlands. BMC Palliat Care.

[42] Howland RH (2009). Effects of aging on pharmacokinetic and pharmacodynamic drug processes. J Psychosoc Nurs Ment Health Serv.

[43] Paque K, Vander Stichele R, Elseviers M, Pardon K, Dilles T, Deliens L (2019). Barriers and enablers to deprescribing in people with a life-limiting disease: a systematic review. Palliat Med.

[44] van Nordennen RTCM, Lavrijsen JCM, Heesterbeek MJAB, Bor H, Vissers KCP, Koopmans RTCM (2016). Changes in prescribed drugs between admission and the end of life in patients admitted to palliative care facilities. J Am Med Dir Assoc.

[45] Wenedy A, Lim YQ, Lin Ronggui CK, Koh GCH, Chong PH, Chew LST (2019). A study of medication use of Cancer and non-cancer patients in home hospice care in Singapore: a retrospective study from 2011 to 2015. J Palliat Med.

[46] McLean S, Sheehy-Skeffington B, O’Leary N, O’Gorman A (2013). Pharmacological management of co-morbid conditions at the end of life: is less more?. Ir J Med Sci.

[47] Goh SSL, Lai PSM, Ramdzan SN (2023). Weighing the necessities and concerns of deprescribing among older ambulatory patients and primary care trainees: a qualitative study. BMC Prim Care.

[48] Rijpstra M, Kuip E, Hasselaar J (2023). The clinical practice of palliative sedation in patients dying from COVID-19: a retrospective chart review. BMC Palliat Care.

[49] Beng TS, Kim CLC, Shee CC (2022). COVID-19, suffering and Palliative Care: a review. Am J Hosp Palliat Care.

[50] Lovell N, Maddocks M, Etkind SN (2020). Characteristics, Symptom Management, and outcomes of 101 patients with COVID-19 referred for Hospital Palliative Care. J Pain Symptom Manage.

[51] Ramos-Rincon JM, Moreno-Perez O, Gomez-Martinez N (2021). Palliative Sedation in COVID-19 end-of-Life Care. Retrospective Cohort Study Medicina (Kaunas).

[52] Stiel S, Krumm N, Schroers O, Radbruch L, Elsner F (2008). Indikationen Und Gebrauch Von Benzodiazepinen auf einer palliativstation [Indications and use of benzodiazepines in a palliative care unit]. Schmerz.

[53] Senderovich H, Gardner S, Berall A, Ganion M, Zhang D, Vinoraj D, Waicus S (2022). Benzodiazepine Use and Morbidity-Mortality outcomes in a geriatric Palliative Care Unit: a retrospective review. Dement Geriatr Cogn Disord 3 March.

[54] Bowers B (2023). BMJ Supportive & Palliative Care.

[55] Mumoli N, Florian C, Cei M, Evangelista I, Colombo A, Razionale G, Moroni L, Mazzone A (2021). Palliative care in a COVID-19 Internal Medicine ward: a preliminary report. Int J Infect Dis.

[56] Sun H, Lee J, Meyer BJ, Myers EL, Nishikawa MS, Tischler JL, Blinderman CD (2020). Characteristics and Palliative Care needs of COVID-19 patients receiving Comfort-Directed Care. J Am Geriatr Soc.

[57] Lovell N, Maddocks M, Etkind SN, Taylor K, Carey I, Vora V, Marsh L, Higginson IJ, Prentice W, Edmonds P (2020). Characteristics, Symptom Management, and outcomes of 101 patients with COVID-19 referred for Hospital Palliative Care. J Pain Symptom Manag.

[58] Wong AK, Demediuk L, Tay JY, Wawryk O, Collins A, Everitt R, Philip J, Buising K (2021). Le B. COVID-19 end-of-life care: symptoms and supportive therapy use in an Australian hospital. Intern Med J.

[59] Alderman B, Webber K, Davies A (2020). An audit of end-of-life symptom control in patients with corona virus disease 2019 (COVID-19) dying in a hospital in the United Kingdom. Palliat Med.

[60] Jackson T, Hobson K, Clare H, Weegmann D, Moloughney C, McManus S (2020). End-of-life care in COVID-19: an audit of pharmacological management in hospital inpatients. Palliat Med SAGE.

[61] Tenge T (2022). Specialist Palliative Care consultations in COVID-19 patients in the ICU-A retrospective analysis of patient characteristics and symptoms at a German University Hospital. J Clin Med.

[62] Clemens KE, Quednau I, Klaschik E (2009). Use of oxygen and opioids in the palliation of dyspnoea in hypoxic and non-hypoxic palliative care patients: a prospective study. Support Care Cancer.

[63] Abernethy AP, McDonald CF, Frith PA (2010). Effect of palliative oxygen versus room air in relief of breathlessness in patients with refractory dyspnoea: a double-blind, randomised controlled trial. Lancet.

[64] Quinn-Lee L, Weggel J, Moch SD (2018). Use of Oxygen at the end of life: attitudes, beliefs, and practices in Wisconsin. WMJ.

[65] Sánchez-Sánchez E, Ruano-Álvarez MA, Díaz-Jiménez J, Díaz AJ, Ordonez FJ (2021). Enteral Nutrition by Nasogastric Tube in Adult patients under Palliative Care: a systematic review. Nutrients.

[66] Good P, Cavenagh J, Mather M, Ravenscroft P. Medically assisted nutrition for palliative care in adult patients. Cochrane Database Syst Rev. 2008, CD006274. 10.1002/14651858.CD006274.pub2 18843710

[67] American Geriatrics Society Ethics Committee and Clinical Practice and Models of Care Committee (2014). American Geriatrics Society Feeding Tubes in Advanced Dementia position Statement. J Am Geriatr Soc.

[68] Anantapong K, Davies N, Chan J, McInnerney D, Sampson EL (2020). Mapping and understanding the decision-making process for providing nutrition and hydration to people living with dementia: a systematic review. BMC Geriatr.

[69] Ying I (2015). Artificial nutrition and hydration in advanced dementia. Can Fam Physician.

[70] Teno JM (2010). Hospital Characteristics Associated with Feeding Tube Placement in nursing home residents with Advanced Cognitive Impairment. JAMA.

[71] Wernli U, Dürr F, Jean-Petit-Matile S, Kobleder A, Meyer-Massetti C (2022). Subcutaneous drugs and off-label use in Hospice and Palliative Care: a scoping review. J Pain Symptom Manage.

[72] Fainsinger RL, MacEachern T, Hanson J, Bruera E (1992). The use of urinary catheters in terminally ill cancer patients. J Pain Symptom Manage.

[73] Pais R, Lee P, Cross S, Gebski V, Aggarwal R (2020). Bladder care in Palliative Care inpatients: a prospective dual site Cohort Study. Palliat Med Rep.

[74] Covinsky KE (2016). Risks Associated with catheters. JAMA Intern Med.

[75] Alsuhail AI, Punalvasal Duraisamy B, Alkhudhair A, Alshammary SA, AlRehaili A. The Accuracy of Imminent Death Diagnosis in a Palliative Care Setting. Cureus. 2020;12(8):e9503. Published 2020 Aug 1. 10.7759/cureus.9503. 10.7759/cureus.9503 PMC7458715 32879825

